# Gut microbiome in gastrointestinal cancer: a friend or foe?

**DOI:** 10.7150/ijbs.69331

**Published:** 2022-06-21

**Authors:** Yang Liu, Yoshifumi Baba, Takatsugu Ishimoto, Xi Gu, Jun Zhang, Daichi Nomoto, Kazuo Okadome, Hideo Baba, Peng Qiu

**Affiliations:** 1 Department of Oncology, Shengjing Hospital of China Medical University, Shenyang, 110004, Liaoning province, China.; 2 Department of Gastroenterological Surgery, Graduate School of Medical Sciences, Kumamoto University, Kumamoto, Japan.; 3 Department of Next-Generation Surgical Therapy Development, Graduate School of Medical Sciences, Kumamoto University, Kumamoto, Japan.; 4 Gastrointestinal Cancer Biology, International Research Center for Medical Sciences, Kumamoto University, Kumamoto, Japan.; 5 Center for Metabolic Regulation of Healthy Aging, Kumamoto University, Kumamoto, Japan.; 6 Department of Anesthesiology, Shengjing Hospital of China Medical University, Shenyang, 110004, Liaoning Province, China.

**Keywords:** gut microbiota, GI cancer, carcinogenesis, chemotherapy

## Abstract

The impact of the gut microbiome on host health is becoming increasingly recognized. To date, there is growing evidence that the complex characteristics of the microbial community play key roles as potential biomarkers and predictors of responses in cancer therapy. Many studies have shown that altered commensal bacteria lead to cancer susceptibility and progression in diverse pathways. In this review, we critically assess the data for gut microbiota related to gastrointestinal cancer, including esophageal, gastric, pancreatic, colorectal cancer, hepatocellular carcinoma and cholangiocarcinoma. Importantly, the underlying mechanisms of gut microbiota involved in cancer occurrence, prevention and treatment are elucidated. The purpose of this review is to provide novel insights for applying this understanding to the development of new therapeutic strategies in gastrointestinal cancer by targeting the microbial community.

## Introduction

The human symbiotic microbial community consists of >100 trillion microorganisms, including bacteria, viruses, fungi and protozoa, which mainly live on the surface of human epithelia, including the skin and digestive and respiratory tracts 1. The gut microbiota is the main components of the human microbial community, which has the largest number of bacteria and the highest diversity compared to the microbiome in other parts of the body. The gastrointestinal (GI) tract is an extension of our natural environment, providing a suitable living environment and rich nutrition for the microbiota. The gut microbiota produces short-chain fatty acids (SCFAs) by metabolizing dietary fiber, synthesizing vitamins B and K 2, metabolizing a variety of compounds such as sterols and exogenous substances, and regulating immune function, which have beneficial effects on the human body 3. Microbes in the GI tract can play a role in the maintenance of host physiological and immune functions and contribute to the pathogenesis of a variety of chronic diseases by interfering with the immune system 4. Numerous risk factors, including pathogens, are thought to be associated with the development of human cancer 5-7. A mechanistic link between commensal gut microbiota and cancer has emerged in recent years, including convincing evidence of the gut microbiome in cancer onset, development and regulation of therapeutic response 8-10. In this review article, we have reviewed the effect of the gut microbiome on GI carcinogenesis and discuss the role and implication of gut microbiota in the treatment of GI cancer. Modulation of the gut microbiome may be used as an adjunct strategy for anticancer therapy. Thus, the genetic background of the patients and lifestyle factors such as diet and exercise also affect the diversity of the intestinal microbiome and ultimately influence cancer occurrence and treatment.

## Gut microbiota and GI cancer carcinogenesis

**Figure 1:**
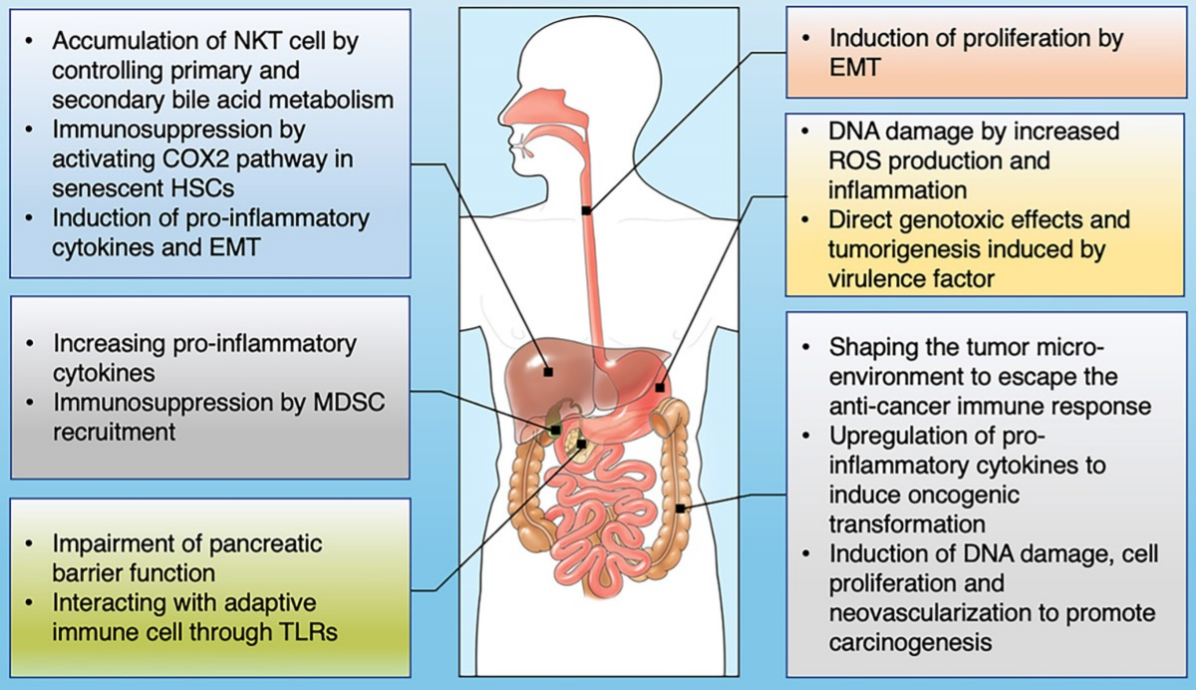
The influence of gut microbiome on the development of GI cancer. Shown are the main mechanisms through which the gut microbiota is proposed to affect the tumorigenesis across GI cancer types, including esophagus, stomach, colon, liver, pancreas and bile duct.

With the rapid development of next-generation high-throughput sequencing (NGS), the role of microbial communities in the host ecosystem has been comprehensively characterized 11, 12. Microbes in the GI tract provide protection and maintain a balance in the host by regulating a large number of basic biological processes 13 (Fig. 1). Microbiome disturbance, known as dysbiosis, is associated with a variety of pathological conditions, such as neurological and behavioral disorders, diabetes, obesity, rheumatic and inflammatory diseases, metabolic syndrome, liver cirrhosis and even various cancers 14, 15. Dysbiosis leads to microecological changes and activates inflammatory factors in the GI mucous membranes, such as the activation of oxidative stress, the release of nitric oxide (NO), the production and secretion of pro-inflammatory cytokines and cyclo-oxygenase 2 (COX-2). Harmful microbial metabolites can affect the normal state of extra-intestinal organs in many ways, with adverse impacts on the gut-brain axis and gut-liver axis 16, 17. With regard to carcinogenesis, it is believed that dysbiosis should be considered as a continuous deviation of host-microbiota from a health-related and homeostatic state for promoting and/or sustaining various cancer phenotypes 18. Well-balanced gut microbiota plays a crucial role in a healthy life, while dysbiosis can have inflammatory consequences that aggravate the development of cancer 19. Numerous preclinical studies have revealed the role of gut microbiota in the occurrence and progression of cancer through different mechanisms.

### Esophageal squamous cell carcinoma (ESCC)

**Figure 2:**
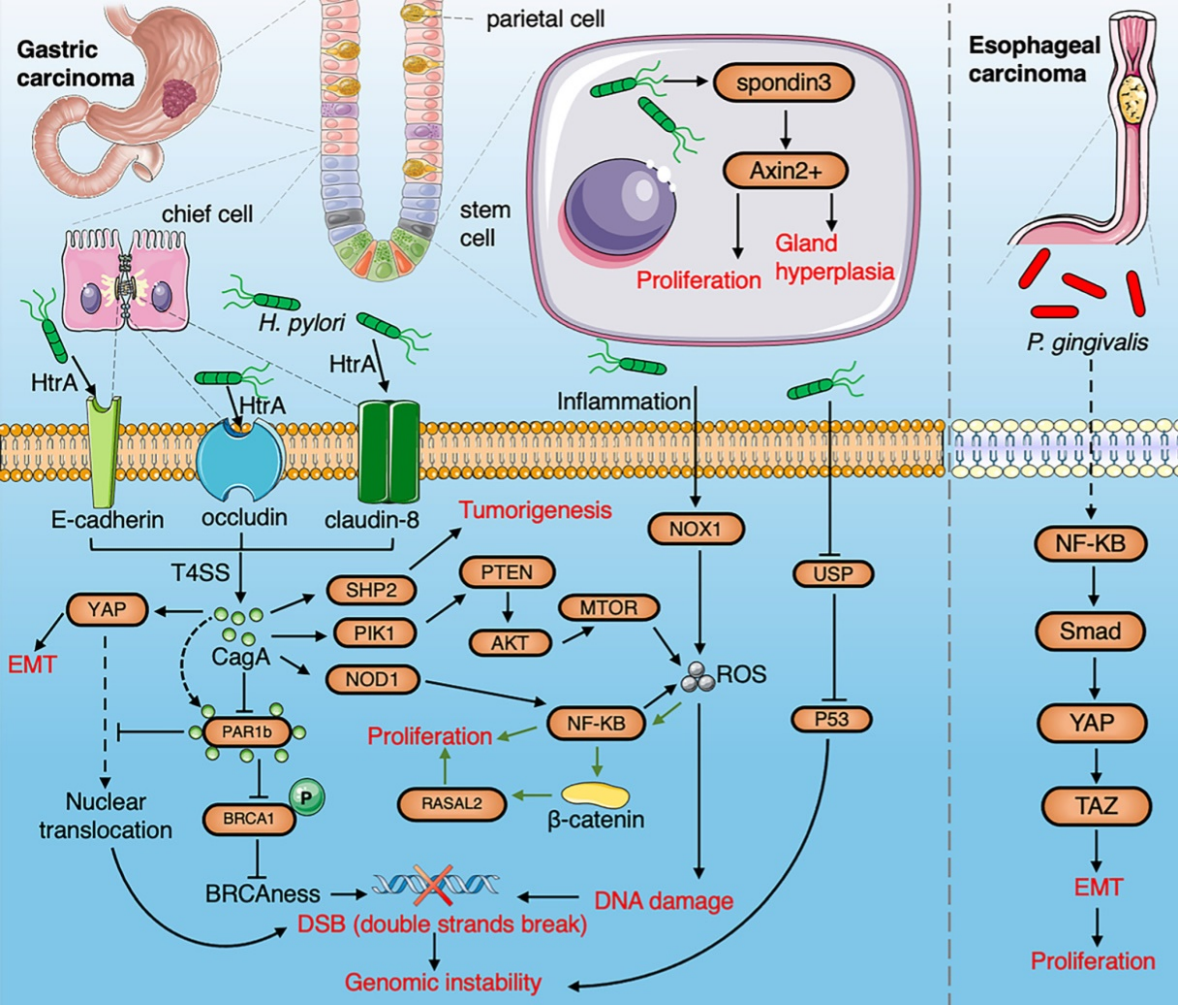
A summarized figure demonstrating the linkage between the gut microbiome and gastric cancer and esophageal cancer. Section 1 Gastric cancer: HtrA protease secreted by* H.pylori* produce CagA protein by cleaving occludin, claudin-8 and E-cadherin. The cagA protein promotes EMT by activating the YAP pathway and inducing tumorigenesis through activation of NF-κB, PTEN, and SHP2 pathways. The production of ROS induced by *H.pylori* via the NF-κB pathway and inflammation contributes to DNA damage.* H. pylori* infection induces gastric stem cells to proliferate and stimulate gland hyperplasia through R-spondin 3 and Axin2. The interaction of CagA and PAR1b induces genomic instability by inhibiting the nuclear translocation of BRCA1 and YAP. The hypermethylation of USF1 by *H. pylori* degrades the p53 proteasome to promote gene instability. *H.pylori*-induced inflammation upregulates NOX1/ROS signaling pathway to promote the stemness of gastric cancer. *H. pylori* promotes the proliferation of cancer cell infection by activating the RASAL2/β-catenin signaling pathway. *Section 2* ESCC*: P. gingivalis* trigger activation of NF-κB to induce EMT through the Smad/YAP/TAZ signaling pathway.

The esophageal mucosa has a large surface area that is located between the oropharynx and stomach with a large number and diverse microbiota. Microbes can easily enter the esophagus by swallowing and reflux. Blackett et al. found a significant increase in the abundance of *Campylobacter* in patients with gastroesophageal reflux disease (GERD) and Barrett's esophagus 20. *Campylobacter* is considered to induce inflammation of the esophageal mucosa, followed by epithelial metaplasia, eventually leading to malignant transformation 21. Elliott et al. found that microbial diversity in esophageal adenocarcinoma (EAC) decreases while the relative abundance of *Lactobacillus fermentum* increases, and some species of *Lactobacillus* are enriched in tumors in about 50% of EAC patients 22. Zaidi et al. found that *Escherichia coli* (*E. coli*) is abundant in EAC. The expression of certain Toll-like receptors (TLR1, 3, 6, 7 and 9) in tumor tissue from a rat model of EAC was also significantly up-regulated 23. The pathogenesis of *Helicobacter pylori* (*H. pylori*) in EAC is still controversial. Etiological studies have shown that *H. pylori* might reduce the incidence of EAC by inhibiting gastric acid secretion to reduce reflux esophagitis, and alter the number of T cells 24. In contrast, *H. pylori* has been demonstrated to induce the occurrence of GERD 25. Several studies have found that *Tannerella forsythia* is associated with a higher risk of EAC, symbiotic *Neisseria* and *Streptococcus pneumoniae* are associated with a lower risk of EAC and enrichment of *Porphyromonas gingivalis* (*P. gingivalis*) is an important risk factor for ESCC 26-29. *P. gingivalis* triggers the nuclear factor (NF)-κB signaling pathway to induce proliferation and metastasis of ESCC cells 30, and it induces epithelial-mesenchymal transformation (EMT) through transforming-growth-factor (TGF)-dependent Smad/YAP/TAZ signaling pathway 31 (Fig. 2). *Fusobacterium nucleatum* (*F. nucleatum*) is associated with the stage of ESCC and poor prognosis and could be used as a biomarker for the outcome of ESCC. It has also been revealed by KEGG enrichment analysis that *F. nucleatum* activates the chemokine CCL20 to promote tumor invasiveness 32.

### Gastric cancer

*H. pylori* infection is one of the major risk factors for the development of gastric adenocarcinoma 33. Among the patients infected with* H. pylori*, the diversity of gastric microbiota is lower than that of healthy people and successful elimination of* H. pylori* from the GI tract using antibiotics reduces the risk of gastric cancer by 75% 34. Colonization of the human gastric mucosa by *H. pylori* affects the plasticity and homeostasis of gastric epithelial cells and promotes the carcinogenesis of epithelial cells 35. HtrA protease secreted by *H. pylori* destroys the protective layer of epithelial cells by cleaving three proteins: closed protein (occludin), tight junction protein-8 (claudin-8) and epithelial cadherin (E-cadherin), and secreted CagA protein reprograms host cells and induces carcinogenesis 36. The stem cell driving signal R-spondin controls the renewal of two types of gastric stem cells after *H.pylori* infection via the Wnt pathway 37. Intragastric colonization of the gut microbiota has been shown to promote *H. pylori*-associated gastric cancer 38, 39. The microbial community in patients with gastric cancer increases nitrosation, which is consistent with increased genotoxicity potential 40. Recent studies have also highlighted that miRNA-mediated regulation and epigenetic modifications, through DNA methylation, are key events in *H. pylori*-induced tumorigenesis, resulting in a stronger carcinogenic activity of the CagA protein 41, 42. The CagA protein of *H. pylori* promotes genetic instability, EMT and carcinogenesis by activating novel CagA‐dependent pathways including YAP 43. CagA-positive *H.pylori* inhibits the partitioning-defective 1b (PAR1b). The interaction of CagA and PAR1b inhibits the nuclear translocation of BRCA1 and YAP, which in turn induces genomic instability 44. *H.pylori* can lead to the hypermethylation of the upstream promoter region of transcription factor (USF1), degrades the p53 proteasome, and promotes the accumulation of gene mutations, thereby accelerating the occurrence of gastric cancer 45. *H.pylori*-induced inflammation upregulates NF-κB through the nicotinamide adenine dinucleotide phosphate oxidase 1 (NOX1)/ROS signaling pathway, thereby promoting the uncontrolled proliferation of gastric epithelial stem cells 46. *H. pylori* infection activates ras protein activator like 2 (RASAL2) by β-catenin to promote the proliferation of cancer cell 47. *H. pylori* also induces methylation of CpG islands 48. Gastric colonization by other intestinal bacteria such as *Peptostreptococcus stomatis*, *Streptococcus anginosus*, *Parvimonas micra* (*P. micra*), *Slackia exigua* and *Dialister pneumosintes* affects the risk of developing gastric cancer 49. Intragastric colonization by the gut microbiota has been shown to promote the occurrence of *H. pylori*-associated gastric cancer 50. Following the eradication of *H. pylori*, other gastric microbes can play a potential role in the development and persistence of gastric precancerous lesions by functional pathways (Fig. 2), which might be used as therapeutic targets for the prevention of gastric cancer 51.

### Hepatocellular carcinoma (HCC)

**Figure 3:**
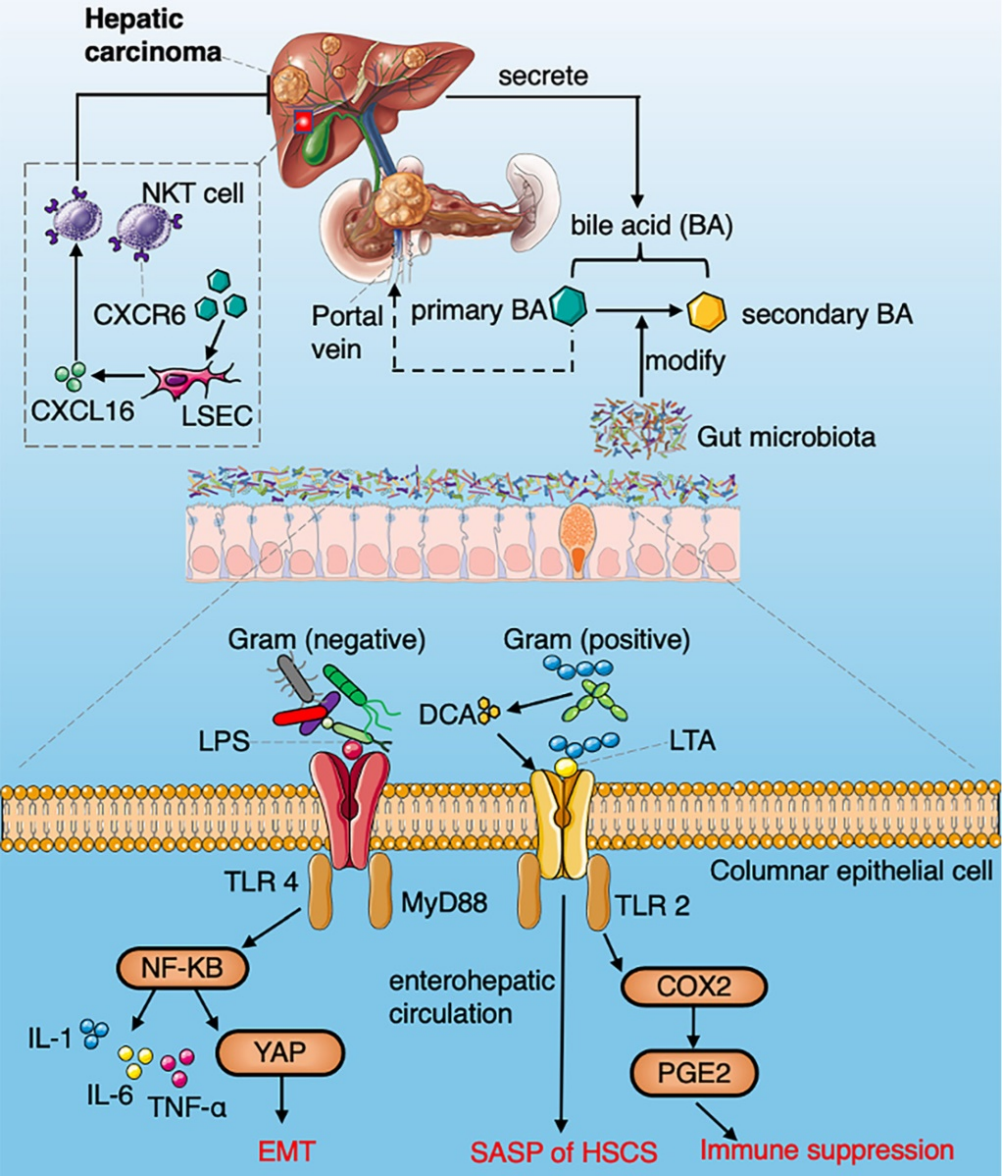
A summarized figure demonstrating the mechanisms of gut microbiome regulating HCC development. Section 1 Commensal bacteria regulate the metabolism of primary and secondary bile acids to control NKT accumulation via CXCL16. Section 2 LPS arising from gram-negative bacteria activate the NF-κB signaling pathway and produce a variety of pro-inflammatory cytokines. LTA in gram-positive bacteria collaboratively with DCA upregulate SASP and COX-2 through TLR2 in HSCs, COX2 mediated PGE_2_ attenuate anti-tumor immunity through PTGER4.

The gut-liver axis composed of the portal system and the biliary system is the anatomical basis for the reciprocal interaction of gut microbiota and liver 52. The dysbiosis and gut leakage induced by various chronic pathogenic factors such as viruses, alcohol and metabolic abnormalities make the liver exposed to the gut microbiota and the metabolites. The liver regulates the host metabolism, immune response and affects intestinal function through bile secretion and enterohepatic circulation 53. Gut microbiota (mainly pathogenic bacteria) related molecular patterns and metabolites, such as deoxycholic acid (DCA) and lipopolysaccharide (LPS), can promote liver inflammation, fibrosis and genotoxicity, activate antiapoptotic signaling pathways and trigger immune responses which contribute to HCC development^54^ (Fig. 3). Therefore, the gut microbiota plays important role in promoting the development of HCC, increasing the abundance of LPS-producing bacteria that activate the NF-κB signaling pathway and producing a variety of pro-inflammatory cytokines (TNF-α, IL-6 and IL-1), leading to liver inflammation and oxidative damage 55. Ren et al. have found that 13 genera including *Gemmiger* and *Parabacteroides* are significantly enriched in patients with early HCC compared with liver cirrhosis patients. Butyrate is a kind of SCFAs produced by the intestinal microbiome, which plays a very important role in maintaining the integrity of intestinal epithelial cells, inhibiting intestinal inflammation and tumorigenesis 56, 57. The decreasing of butyrate-producing bacteria leads to the destruction of the intestinal mucosa and tumorigenesis of HCC 58. Activation of TLR4 induced by LPS can enhance the invasiveness of HCC and induce EMT 59. It has been shown that primary bile acid enhances the expression of chemokine CXCL16, which increases the accumulation of natural killer (NK) T cells, resulting in inhibition of HCC 60. Lipoteichoic acid (LTA) in gut microbiota collaboratively with DCA can upregulate the expression of senescence-associated secretory phenotype (SASP) and COX-2 through TLR2 in hepatic stellate cells, while COX-2-mediated prostaglandin E_2_ (PGE_2_) inhibits anti-tumor immunity through prostaglandin EP4 receptors 61. *Clostridium* metabolizes bile acid into DCA, thus increasing the serum level of DCA in HCC 62. Yoshimoto et al. have proved that inhibiting DCA production or modulating gut microbiota efficiently prevented the tumorigenesis of HCC 63. Diet fat, cholesterol, fiber or carbohydrate could modulate gut microbiome composition by a number of metabolic pathways to contribute to the development of non-alcoholic steatohepatitis (NASH) and non-alcoholic fatty liver disease (NAFLD)-HCC 64. Dysbiosis caused by a high-fat diet can affect the expression of specific miRNA in HCC 65. Dietary cholesterol induces an increase in taurocholic acid and a decrease in indole propionic acid which drive the occurrence of NAFLD-HCC 66. It was found that the intake of soluble cellulose can lead dysbiosis and induce cholestatic liver cancer 67. Dietery fructose was found to promote the liver lipid synthesis and re-shape the microbial composition with an increased abundance of *Bacteroides* in NASH which increases the risk of NAFLD-HCC 68, 69. Liu et al. investigated the differences in gut microbes between hepatitis B virus (HBV)-related HCC (B-HCC) and non-HBV non-hepatitis-C-virus-associated HCC (NBNC-HCC). They found a greater increase in pro-inflammatory bacteria (*Escherichia shigella, Enterococcus*) in the feces of NBNC-HCC patients, and decreased levels of *Faecalibacterium, Ruminiclostridium and Ruminococcus*, which led to a decrease in the potential of anti-inflammatory SCFAs.

Compared with NBNC-HCC patients, B-HCC patients exhibit markedly contrasting results in terms of bacterial composition and biological pathways, which suggest that modifying specific microbes could provide therapeutic benefits for B-HCC and NBNC-HCC 70. *E. coli* impaired and penetrated the gut vascular barrier, and colonized the liver to recruit immune cells, thus facilitating the formation of the premetastatic niche and promoting liver metastasis 71. The enrichment of *F. nucleatum* reduced the diversity of gut microbiota in mice leading to dysbiosis of intestinal microbiota. *F. nucleatum* significantly increased the serum level of pro-inflammatory cytokines and reduced the cytotoxicity of immune cells to promote liver metastasis in mice 72. Current data from preclinical and clinical studies pointing to the targeting gut microbiota and gut-liver axis can be used to monitor and prevent the progression of HCC.

### Colorectal cancer (CRC)

**Figure 4:**
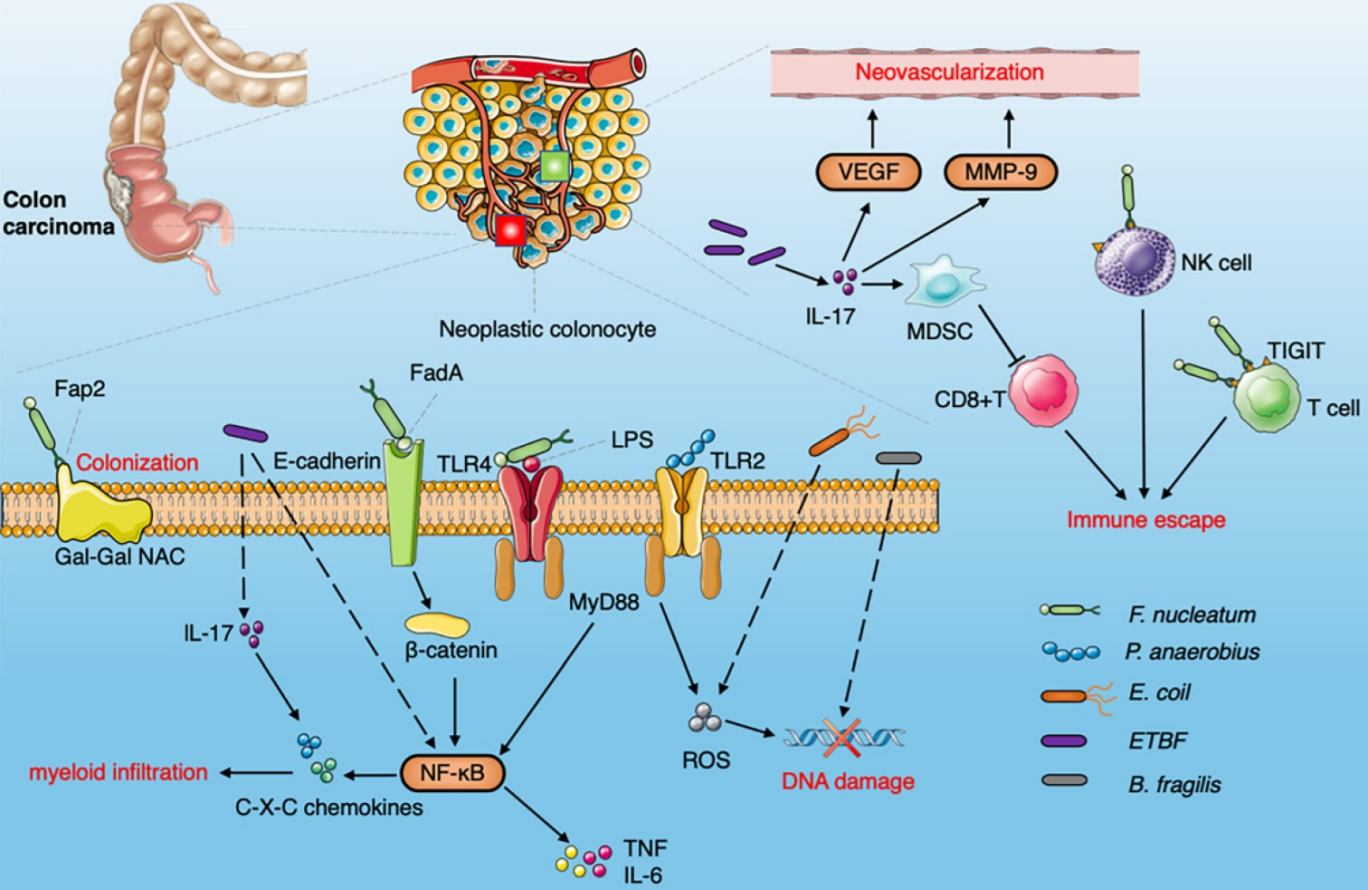
** Diagram summarizing the oncogenic interaction between the gut microbiome and CRC.** The Fap2 protein combined with Gal-GalNAc is enriched on the surface of CRC cells to promote the colonization of *F.nucleatum. F. nucleatum* binds to E-cadherin on intestinal epithelial cells through FadA , activates the β-catenin signaling pathway, induces NF-κB pathway activation and upregulates pro-inflammatory cytokines, produces Fap2 to bind to TIGIT receptors on NK cells and other TILs. *F.nucleatum* activate NF-κB pathway via TLR4 and MYD88 to promote cell proliferation and invasion. *Peptostreptococcus anaerobius* increase the level of ROS via TLR2 and/or TLR4 signaling pathways. ETBF induce IL-17 and NF-κB pathway to enhance the production of C-X-C chemokine. MDSCs recruited by ETBF via IL-17 to inhibit the activity of cytotoxic CD8 + T cells. ETBF enhances the expression of MMP9 and VEGFA to induce neovascularization through IL-17.* E. coli* can also increase the level of ROS and promote the accumulation of spontaneous mutations. *B. fragilis* induces DNA damage.

CRC is one of the most common forms of malignancy with high morbidity and mortality 73. Although the main gut microbiota involved in CRC has not been fully determined, the importance of *F. nucleatum, E. coli* and *Bacteroides fragilis* (*B. fragilis*) in CRC has been proved, which could promote tumor progression by inducing DNA damage and other signaling pathways 74 (Fig. 4). The Fap2 protein can combine with the Gal-GalNAc enriched on the surface of CRC cells to facilitate *F. nucleatum* colonization in the host cell 75. *F. nucleatum* binds to E-cadherin of intestinal epithelial cells through FadA, activates the β-catenin signaling pathway, induces NF-κB pathway activation, upregulates proinflammatory cytokines (TNF-α, IL-6, IL-8 and IL-1β), and induces Fap2 to bind to TIGIT receptors on NK cells and other tumor infiltrating lymphocytes (TILs) thus promoting cancer progression and immune escape 76, 77. It has the capacity to activate the NF-κB pathway via TLR4 and MyD88 to promote the proliferation and invasion of CRC 78. Bullman et al. have demonstrated that *F. nucleatum* is enriched in primary lesions of CRC, and can be found in liver metastasis 79. *B. fragilis* and *E. coli* strain psk+ penetrate the mucous layer on the surface of the colon, which usually performs important barrier functions 80. The toxins released by *E. coli* and *B. fragilis* damage double-stranded DNA and promote cellular inflammation and oncogenic mutation 81, 82. EspF, a targeted mitochondrial effector protein secreted by enteropathogenic *E. coli*, removes DNA mismatch repair proteins from the host, thereby reducing DNA repair activity and increasing mutation rates in patients with CRC. *E. coli* also increases the level of reactive oxygen species (ROS) and promotes the accumulation of spontaneous mutations 83.* Peptostreptococcus anaerobius* (*P. anaerobius*) is enriched in CRC and promotes carcinogenesis by increasing the level of ROS via TLR2 and/or TLR4 signaling pathways in mice 84. Based on an analysis of 526 metagenomic samples from a multinational cohort, seven bacteria (*B. fragilis, F. nucleatum, Porphyromonas asaccharolytica, P. micra, Prevotella intermedia, Alistipes finegoldii, and Thermanaerovibrio acidaminovorans* enriched in CRC were identified among different populations. The potential bacterial diagnostic markers were robust among these populations. The bacteria enriched in CRC were associated with LPS-induced inflammation and energy biosynthesis pathways 85. Yachida et al. have shown that the abundance of *Atopobium parvulum* and *Actinomyces odontolyticus* increases in early CRC, and enrichment of these two bacteria promotes the occurrence of CRC, which suggests that it could be used as a biomarker for early detection of CRC 86. Enterotoxigenic *B. fragilis* (ETBF) has been found to induce IL-17 and trigger the NF-κB pathway to enhance the production of C-X-C chemokine. Myeloid-derived Suppressor Cells (MDSCs) are recruited by ETBF infection which inhibits the activity of cytotoxic CD8 + T cells and ETBF induces the expression of matrix metalloproteinase 9 (MMP9) and vascular endothelial growth factor A (VEGFA) thus triggering CXCL1 and CXCL2 via IL-17 87, 88 (Fig. 4). Oral administration of CRC-associated *Streptococcus gallolyticus* in mice with dextran-sodium-sulfate-induced CRC results in increased tumor burden, selective recruitment of CD11b + myeloid cells and increased expression of cytokines (including IL-6 and IL-8) 89. Gut microbiota is also involved in regulating the expression of plasma membrane transporter SLC5A8, cell-surface G-protein-coupled receptor GPR109A and GPR43 in the colon, enhancing the memory potential of CD8+ cells to manipulate the immune response and tumor cell proliferation and apoptosis 90-92. Therefore, the strategy of promoting transformation from a “cold tumor” to a “hot tumor” (characterized by infiltration of CD8+ T cells) by modulating the gut microbiota will be a novel, timely and interesting therapeutic approach.

### Cholangiocarcinoma

The occurrence of cholangiocarcinoma is related to gut dysbiosis 93. Chng et al. found that the colonized flora in cholangiocarcinoma tissue was significantly different from paracancerous tissue and normal liver tissue, and *Pseudomonadaceae* is enriched in tumor lesions. The abundance of *Bifidobacteriaceae, Enterobacteriaceae* and *Enterococcaceae* is associated with the development of *Opisthorchis viverrini* (*O. viverrini*) cholangiocarcinoma 94. Di Carlo et al showed that the patients with cholangiocarcinoma had a poor prognosis when infected with *Klebsiella pneumonia* (*K. pneumonia*) 95. Jia et al. showed that *Actinomyces, Lactobacillus, Peptostreptococcaceae* and *Alloscardovia* were significantly enriched in intrahepatic cholangiocarcinoma. The enrichment of glycoursodeoxycholic acid and tauroursodeoxycholic acid (TUDCA) plasma-stool ratios (PSRs) can be used as biomarkers to distinguish intrahepatic cholangiocarcinoma and HCC. The increase of *Ruminococcaceae* was related to the concentration of plasma IL-4, IL-6 and Chenodeoxycholic acid (CDCA) in intrahepatic cholangiocarcinoma 96. Avilés-Jiménez et al. reported that *Nesterenkonia* was decreased in extrahepatic cholangiocarcinoma, while the enrichment of *Actinomyces, Novosphingobium, Methylophilaceae, Fusobacterium, Prevotella* and *H. pylori* increased compared with normal biliary tract 97. *H. pylori* has been proved to promote EMT of bile duct epithelial cells, which is related to the occurrence of cholangiocarcinoma 97. Gram-negative bacteria and LPS induce CXCR2+ polymorphonuclear-MDSC accumulation through TLR4-dependent CXCL1 production to control hepatocytes to form an immunosuppressive microenvironment, thereby promoting cholangiocarcinogenesis 98.

### Pancreatic ductal adenocarcinoma (PDAC)

Researchers have gradually gained an in-depth understanding of PDAC, and there is evidence that the occurrence, development and treatment of PDAC are related to the gut microbiota. Fan et al. have shown that enrichment of *P. gingivalis* and* Actinobacillus actinomycetemcomitans* is associated with a high risk of PDAC 99. Patients with high antibody levels to *P. gingivalis* had a more than a two-fold increased risk of developing PDAC compared with patients with normal antibody levels 100. Thirteen phyla of bacteria were found in PDAC tissues, mainly including *Proteobacteria* (45%),* Bacteroidetes* (31%) and *Firmicutes* (22%) 101. At present, there are several mechanisms for the bacteria to enter the pancreas from the digestive tract, such as the portal circulation, mesenteric lymph nodes and the lower digestive tract. Farrell and colleagues have demonstrated that *Neisseria elongata* and *Streptococcus mitis* were significantly decreased in PDAC patients compared with healthy people 102. In a mouse model, bacteria were involved in the accelerated development of PDAC, which might be mediated by bacterial metabolites. Another possible mechanism is that bacteria may transfer from the intestinal or oral cavity to the pancreas, accompanied by impaired pancreatic barrier function, and become colonized in the pancreas to reset immune tolerance and promote the progression of PDAC. In a small number of patients with long-term survival of PDAC, the gut microbiota in the tumor were significantly more diverse than that in the tumors of short-term survival patients, while *Pseudoxanthomonas*, *Saccharopolyspora, Streptomyces* and* Bacillus Clausii* (*B. Clausii*) were significantly enriched in tumors of long-term survival patients. The number of mature CD8+ T cells and granular B cells in long-term survival patients was significantly more than that in short-term survival patients, which was positively correlated with the amounts of* Pseudoxanthomonas, Saccharopolyspora, Streptomyces* in the gut. The bacteria in these tumors may promote an anti-tumor immune response via recruiting and activating of CD8+ T cells, and fecal microbiota transplant (FMT) from long-term survivors promotes an immune response and suppresses tumors in mouse models by changing the tumor microbiome 103. Preclinical models have shown that intestinal flora could induce transcriptome changes, interact with adaptive immune cells, and induce immunosuppression of PDAC cells through TLR2 and TLR5 pathways in tumor-associated macrophages (TAMs), which plays an important role in the occurrence and development of PDAC 101, 104, 105.

**Figure 5:**
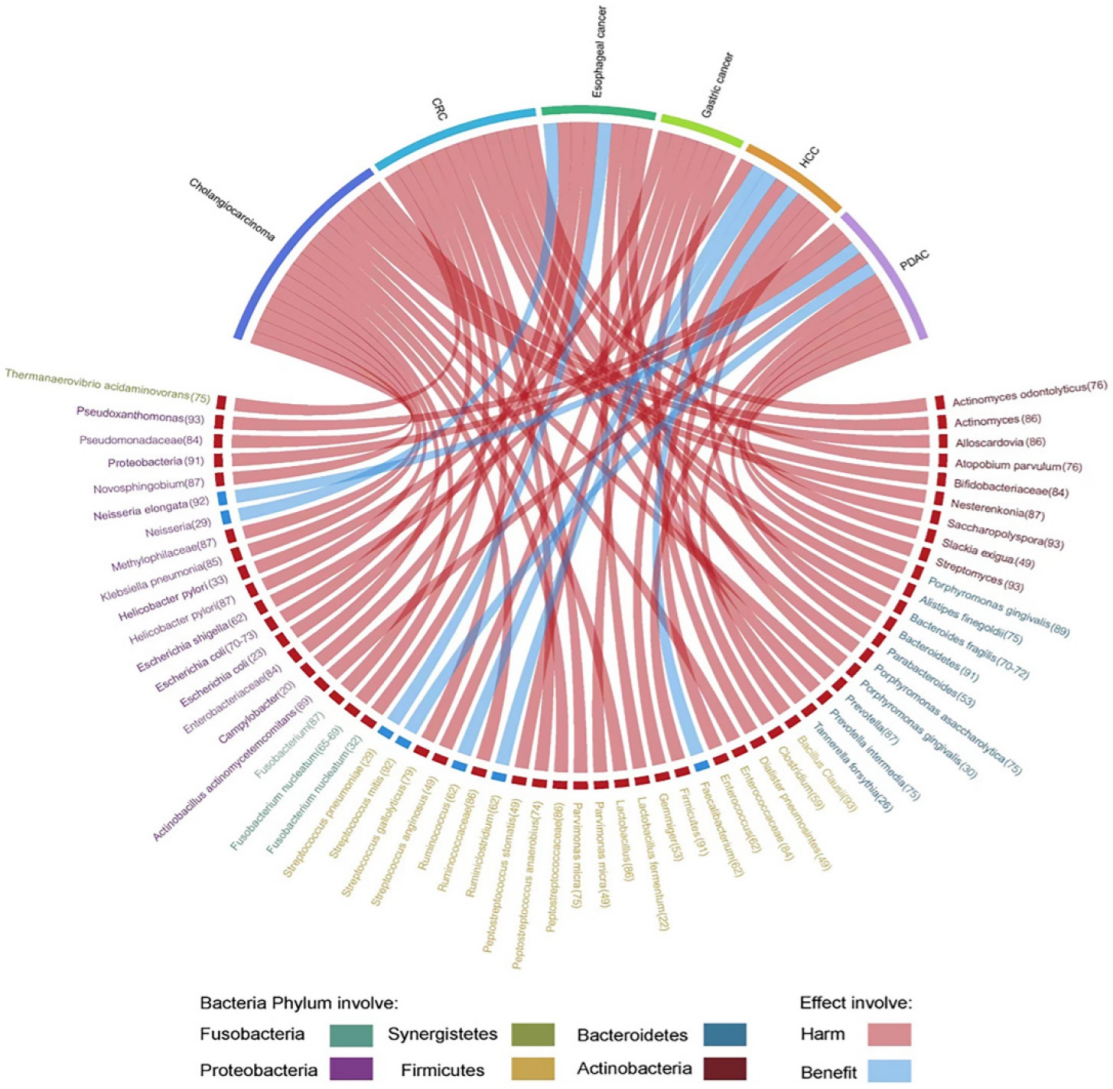
Evidence of the gut microbiota enriched in GI cancer playing a crucial role in carcinogenesis. Circos plots illustrating the correlation of bacteria with tumorigenesis in GI cancer. The red ribbons represent the harmful effects of bacteria on GI cancer development. The blue ribbons represent the beneficial effects of bacteria on GI cancer development. The causality of the microbiota in GI cancer has not yet been fully elucidated. Different taxa are divided into six groups and colored by their phylum. Numbers indicate the references that highlight these relationships.

Herein, we highlight the effect of gut microbiota across the continuum of GI cancer and discuss the correlation of bacteria with tumorigenesis by circos plots (Fig. 5), however, subtle complexities exist, and many questions remain concerning the potential for exploiting the gut microbiota that regulate key mechanisms in carcinogenesis.

## Gut microbiota and therapies for GI cancer

### Gut microbiota in chemotherapy for GI cancer

The gut microbiota regulates host responses to chemotherapeutic drugs by promoting drug efficacy, influencing anticancer effects and toxicity (Table 1) 106. The interaction between gut microbiota and chemotherapeutic drugs can be manipulated by the TIMER mechanistic framework through translocation, immunomodulation, metabolism, enzyme degradation, and ecological variation 107. Yamamura et al. have found that the DNA of intratumoral *F. nucleatum* is associated with the response to neoadjuvant chemotherapy in ESCC patients 108. Liu et al. have found that *F. nucleatum* is an intracellular bacteria that survives in ESCC cells and confers chemoresistance via autophagy 109. *F. nucleatum* targets the TLR4/MyD88 signaling pathway and reduces expression of miRNA-4802 and miRNA-18a, activating autophagy and upregulating expression of autophagy-related genes, which induces chemoresistance in CRC. It has been found that *F. nucleatum* infection reduces chemosensitivity to 5-FU by regulating baculoviral IAP repeat containing 3 (BIRC3) via the TLR4/NF-κB signaling pathway in adjuvant chemotherapy of CRC patients 110, 111. Gemcitabine can be metabolized into inactive 2',2'-difluorodeoxyuridine by* Mycoplasm hyorhinis* through CDDL gene in a mouse CRC model. Furthermore, it has been shown that most of the bacteria in PDAC belong to the *Gammaproteobacteria*, which have the CDDL gene that metabolizes gemcitabine, and this effect can be antagonized by ciprofloxacin 112. A variety of commensal microbiota can affect the efficacy of conventional chemotherapy by modulating the tumor microenvironment. The absence of *Lactobacillus* decreases the cytotoxicity of oxaliplatin by reducing the production of ROS 113. Cyclophosphamide (CTX) induces its anticancer effect by interfering with various immune signaling cascades. A preclinical study has found that CTX-induced immune activation requires the participation of some bacterial species such as *Enterococcus hirae* (*E.hirae*). CTX induces the bacteria to translocate to lymph nodes and the spleen to stimulate the host immune response. Subsequent studies have also found that* E. hirae* and* Barnesiella intestinihominis* are necessary for the anti-tumor effect of CTX 114, 115. Irinotecan is converted into its active form (SN-38) through cleavage of the side chains of carboxylesterase in plasma, intestinal mucosa, liver and tumor cells. Liver UDP-glucuronosyltransferase (UGT)A1 and UGT1A9 and extrahepatic UGT1A7 play a major role in the detoxification of SN-38. The inactive form of bile secretion in the intestinal cavity, SN-38G, is transformed into active metabolite SN-38, by β-glucuronidase produced by gut microbiota. Intestinal mucosal injury and diarrhea are directly caused by irinotecan 116. Butyrate could promote the efficacy of oxaliplatin by modulating the antitumor cytotoxic CD8+ T cell responses via activating the IL-12 signaling pathway 117. A prospective study suggested that the gut microbiota may be used as potential biomarkers for predicting the response to neoadjuvant chemoradiotherapy of patients with locally advanced rectal cancer (LARC) 118. Further baseline analysis of samples before neoadjuvant radiotherapy and chemotherapy found that some bacteria related to SCFA metabolism were significantly enriched in the LARC neoadjuvant chemotherapy response group, while *Clostridium* was significantly enriched in the non-response group. In addition, the beneficial microflora of the response group was more enriched than that of the non-response group, while the pathogenic bacteria of the non-response group were more enriched than the response group 119.

#### Gut microbiota in radiotherapy for GI cancer

The mechanism of intestinal microflora regulating radiotherapy response is still unclear. Radiotherapy can promote tumor cytotoxicity and the systemic immune response regulated by the immune system 120. Because the gut microbiota participates in the regulation of immunogenic cytotoxicity in traditional anticancer therapy strategies, it may also play a similar role in radiotherapy-mediated immune responses (Table 1). The gut microbiota of patients and experimental mice receiving radiotherapy is destroyed, which could cause diarrhea and colitis, partly mediated by IL-1β 121. Radiotherapy can also lead to intestinal cell apoptosis and destruction of intestinal barrier function, these changes regulate intestinal immune response, leading to intestinal inflammation 122. The low diversity of the gut microbiota is related to late radiation-induced bowel disease, and the high enrichment of *Roseburia*, *Clostridium IV*, and *Faecalibacterium* was significantly related to radiation enteropathy, which suggests that the microbiota influence susceptibility to GI adverse effects after radiotherapy 123. The intake of probiotics can inhibit radiation-induced cell injury 124. *Lactobacillus rhamnosus* can induce mesenchymal stem cell pre-migration through the TLR2 pathway, repair the radiation-damaged intestinal mucosa, and protect the normal intestinal recess, but it has no protective effect on transplanted tumor tissue 125. Long-term surviving mice exposed to high doses of radiation have unique microbial characteristics, in which increased abundance of *Lachnospiraceae* and *Enterococcaceae* plays a radiation protective role in reducing hematopoietic and GI tissue damage after radiation. Further studies have shown that SCFAs (especially propionate) and specific tryptophan metabolites produced by the microbiota mediate the radioprotective effect of the microflora. The microbiome and its metabolites promote hematopoiesis and intestinal injury repair, thus, helping the host resist radiation-induced injury and death 126. FMT can improve the survival rate of irradiated mice and alleviate the radiation-induced damage 127. 3-propionic acid derived from FMT is a key metabolite to decrease radiation-induced intestinal toxicity 128. Commensal bacteria can regulate host responses to ionizing radiation and repair the damage induced by radiation. A better understanding of the non-targeted mechanism of radiotherapy and the regulation of bacteria is of inestimable value for improving the effectiveness of radiotherapy, reducing the adverse outcomes of radiotherapy in cancer, and optimizing the treatment of patients exposed to radiotherapy. These findings provide a potential therapeutic target for alleviation of radiation-induced injury and reduction of the adverse effects of radiotherapy in cancer.

#### Gut microbiota in ICIs for GI cancer

The influence of the gut microbiota goes beyond the GI tract itself and affects human immune cell dynamics 129, 130. In addition to the direct regulation of bacteria, bacterial metabolites can be translocated from the intestinal cavity to the lamina propria of the intestinal mucosa, affecting the expression of host immune-related genes 131. LPS and peptidoglycan are important components of the outer membrane of gram-negative bacteria. They induced intestinal immunoregulation by activating host TLRs, which are mainly expressed by intestinal epithelial cells and dendritic cells (DCs). TLRs are actively involved in mediating T cell responses to tumor cells 132-134. Studies have revealed that the gut microbiota plays an important role in modulating the therapeutic response to ICIs 135-137. *B. fragilis* contributes to the maturation of regulatory cells (Tregs) and secretion of anti-inflammatory cytokine IL-10 by activating TLR2-dependent plasmacytoid dendritic cells 138, 139. *Bifidobacterium* stimulates the production of anti-CD47 antibodies by activating the STING signaling pathway, which might alter the tumor microenvironment to achieve the effect of immunotherapy 140. It also can alleviate Checkpoint inhibitors (ICIs)-associated colitis by modulating gut microbiota composition and inhibiting Tregs cell by IL-10 141. Combined with anti-programmed death ligand 1 (PD-L1) therapy, oral *Bifidobacterium* can enhance the function of DCs, promote the initiation and accumulation of CD8 + T cells, and interact with the tumor microenvironment 142. Patients with advanced GI cancer who received anti-PD-1/PD-L1 therapy were recruited in a comprehensive analysis study. The ratio of *Prevotella*/*Bacteroides* in the feces increased, which was associated with prolongation of progression-free survival, and the relative abundance of *Prevotella, Ruminococcaceae, and Lachnospiraceae* in the responders were higher than that in the non-responders 143. The differential functional pathways of the gut microbiota, including nucleoside biosynthesis, lipid biosynthesis, glucose metabolism, and abundance of SCFAs, were related to the different clinical responses to ICI therapy. The propionate increases the differentiation and function of Tregs in the gut^144^. Tregs remodel a proinflammatory state to an anti-inflammatory system by producing anti-inflammatory cytokines in TME. The propionate causes Foxp3 + Tregs to produce IL-10, and through the GPR43 signaling pathway, inhibiting histone deacetylase (HDAC) activity to prevent colitis. The butyrate promotes the production of Foxp3+ Tregs in peripheral tissues by inhibiting HDAC. *Clostridium*-produced butyrate acetylates the Foxp3 gene promoter histone H3 and accelerates Foxp3+ Tregs differentiation 145. SCFA producing bacteria (*Eubacterium, Lactobacillus and Streptococcus*) were positively associated with favorable outcomes of ICI therapy 143. Kenya et al. isolated 11 bacterial strains from the feces of healthy donors. A combination of commensal bacteria can enhance the immune response against infection and cancer by increasing the level of CD8+T cells in a mouse model of CRC 146. Cytotoxic T lymphocyte-associated antigen (CTLA)-4 blockade has been successfully used in tumor immunotherapy. The efficacy is influenced by the composition of the microbial community (*B. fragilis, Bacteroides thetaiotaomicron and Burkholderiales*), which highlights the important role of bacteria in the efficacy of CTLA-4 blockade 147. Gut microbiota controls the synthesis of bile acid metabolites, thereby regulating the amount of intestinal RORγ+ Tregs and regulating intestinal homeostasis^148^. Another study identified two novel secondary bile acids (ω-MCA and isoDCA) that are potent in inducing Tregs differentiation *in vitro*. The isoDCA interacts with farnesoid X receptor (FXR) in dendritic cells. The interaction of FXR enhances the Tregs-inducing effect to inhibit the occurrence of CRC 56. The metabolite Inosine derived from *Bifidobacterium pseudolongum* promotes Th1 cell differentiation and enhances the therapeutic effect of ICIs, and this process is mediated by T-cell specific adenosine A2A receptor signaling in a mouse model of CRC 149.

Current studies have confirmed that the gut microbiota has the potential to regulate the response to ICI therapy, which implies that targeting the gut microbiota is crucial for ICI therapy (Table 1).

#### Modulation of gut microbiota for GI cancer

Targeted bacterial modulations such as FMT, probiotics, prebiotics and indirect metabolites modulation by dietary and engineered bacteria have shown the potential to optimize cancer treatment and are expected to be used in personalized medicine, while fungi, yeast, viruses, and archaea are gradually emerging in the impact of GI cancer treatment.

FMT involves the transfer of functional microbiota from healthy donor feces into a patient's GI tract, rebalancing the intestinal microflora, repairing the intestinal mucosal barrier, regulating the body's immunity and inflammatory response, and providing a potential strategy for enhanced cancer therapy 103, 150. FMT can improve the efficacy of tumor immunotherapy 19, 151 and relieve the adverse side effects of cancer treatment. One case report showed that two cancer patients had significantly abrogated colitis caused by ICIs after FMT. Preliminary data analysis has shown that the application of healthy gut microbes can eliminate ICI-associated colitis, restore the gut microenvironment and increase the density of Tregs in the colonic mucosa. FMT has been shown to have a positive therapeutic impact on immune-related adverse events 152. There are many controversial views about FMT in cancer 153, 154, therefore, more research and clinical data are needed to demonstrate the safety and effectiveness of FMT. In order to ensure that the benefits outweigh the risks, future research should detect harmful bacteria such as multidrug-resistant bacteria in the donated feces. Although at the present we cannot be certain about what the composition of a healthy intestinal microflora should be, it is possible to identify the beneficial gut microbiota and study them in detail. This will allow us to determine the specific bacterial composition in the fecal donors, which is helpful for more detailed and in-depth studies of beneficial therapeutic responses. More active cooperation among basic, translational and clinical research and epidemiological analysis will bring many new opportunities and challenges to the potential exploration of FMT in the field of cancer treatment 155. In addition, due to the short application time of this technology and the lack of long-term safety data, it is important to closely track and carefully record the wellbeing of patients after FMT. Therefore, we also need high-quality clinical data from properly designed trials to further study the practicability and effectiveness of FMT.

Probiotics are involved in modulating the gut microbiota, enhancing the integrity of the intestinal barrier, and inhibiting the growth of pathogenic bacteria 156. A prospective intervention study in patients with CRC has shown that the use of probiotics containing *Lactobacillus acidophilus* and *Bifidobacterium lactobacillus* have a potential role to be a promising supplementary for the prevention and treatment of CRC. Probiotics increase the number of butyrate-producing bacteria (such as* Faecalibacterium* and *Clostridiales* spp) and reduce the number of CRC-related genera (including *Clostridium* and *Streptococcus*) 157. Some Researchers have designed a probiotic therapy that may improve the safety of tumor immunotherapy, including immunotherapy targeting PD-L1 and CTLA-4. The probiotic drugs are released by bacteria and attack the tumor, which promotes the immune response and eventually leads to tumor regression 158. Although probiotics are generally safe, their administration in immunocompromised cancer patients may have a potential risk of opportunistic infections and conferment of antibiotic resistance. In areas such as cancer and immune diseases 159, there is no doubt that the controversy about probiotics will continue, and individualized probiotics may be the treatment strategy of the future.

Prebiotics are indigestible food ingredients that can promote the growth of probiotics. The chemopreventive properties of prebiotics are due to the production of SCFAs, which enhance host immunity 160. Recent studies have shown that two prebiotics, mucin and inulin, can produce different bacterial populations, suggesting that the different activity modes mediated by the two prebiotics stimulate the emergence of anti-tumor immunity in mice 161, 162. Prebiotic spores (spore-dex) may be prepared by host-guest reaction between commercial *Clostridium butyricum* (*C. butyricum*) and chemically modified probiotic dextran. Recent research has shown that spore-dex is specifically enriched in the tumor lesions after oral administration. The dextran is fermented by* C. butyricum* to produce anticancer SCFAs in CRC lesions 163.

Gut microbiota-derived metabolites can alter cellular metabolism and gene regulation, thereby positively impacting the efficiency of tumor therapy, particularly SCFAs can be used as predictive biomarkers for tumor immunotherapy 1. Industrialization, westernization, and food refining have led to dysbiosis which impairs SCFAs production. One study showed that volunteers were fed a high-protein, low-carbohydrate, and low-fiber diet resulting in low levels of butyrate produced bacteria (*Roseburia* and *Eubacterium rectale*), a lower proportion of butyrate in fecal SCFAs, and reducing of the intestinal free phenolic acid 164. Fermentation of dietary fiber from fruits, vegetables and grains is recommended during therapy which might be supposed to affect the outcome of cancer treatment 165.

By modifying the *E. coli* MG1655 expressing nitric oxide synthase to bind carbon nitride (C3N4) on its surface, the photoelectron produced by carbon nitride can be transferred to *E. coli* under light irradiation, so that *E.coli* can better enrich the tumor lesions. Photo-controlled bacterial metabolite therapy (PMT) can inhibit 80% of tumor growth in CRC 166. Niobium carbide (Nb2C)/Au nanocomposites and phototherapy enable “chemical” and “physical” regulation of bacteria, and synergistic microbiota regulation can alter the abundance and diversity of the intratumoral microbiota and disrupt metabolic pathways in the TME. The combination of bacterial manipulation and anti-tumor necrosis factor-α (TNF-α) drugs can synergistically alleviate bacterial-induced inflammation, meanwhile disrupt the metabolism of intratumoral flora to reverse drug resistance, and significantly enhance the response of cancer cells to phototherapy 167.

Intratumoral fungi in pancreatic cancer, especially *Malassezia*, may be highly correlated with the pathogenesis of PDA. Anti-*Malassezia* treatment reduces pancreatic cancer incidence by 40% in mice 168. Fungus stimulates PDAC cells to secrete IL-33, recruits and activates type 2 innate lymphocytes (ILC2), and promotes tumor progression. IL-33 or antifungal drugs may be new targets for pancreatic cancer therapeutic targets 169. A Fungi-based acetaldehyde generator was prepared by modifying alcohol dehydrogenase-loaded metal-organic framework nanocarriers onto the surface of *Saccharomyces cerevisiae* (S. *cerevisiae*), which can target the TAMs by mannose, induce tumor cell apoptosis and promote TAMs polarization to anti-tumor phenotype, and further enhance macrophage-mediated immunotherapy by combining anti-CD47 antibody, significantly inhibit the growth of CRC cell 170. After intratumoral injection, yeast-derived nanoparticles can be effectively recognized by dendritic cells, and can effectively migrate to the lymphatic drainage area, so as to reverse the immunosuppressive microenvironment and inhibit tumor growth. The combination of yeast-derived nanoparticles and PD-L1 antibody has shown a superior antitumor effect in CRC. It can not only effectively eliminate *in situ* tumor, but also inhibit the growth of distal spreading cancer cells 171.

Oncolytic viruses have been proven to be an ideal therapeutic approach to enhance the response of ICIs 172. The mechanisms of oncolytic viruses include carcinolysis, TME modulation, TILs recruitment, angiogenesis and initiation of immune response. Oncolytic viruses are able to enhance immunogenic cell death, which further leads to the recruitment of innate immune cells to form tumor-specific T cells 173. Bacteriophages have the ability to modulate immunity and kill specific bacteria, and the use of phages may become a precise treatment plan that can target “cancer-promoting bacteria” 174. Bacteriophages loaded with silver nanoparticles selectively eliminate F. *nucleatum*, providing a favorable microenvironment for tumor immunotherapy 175.

There are growing evidence that archaea are involved in human cancers 176. Archaea produces a variety of metabolites by utilizing unique metabolic pathways, and cancer-related metabolites (such as polyamines and SCFAs) of archaea are prevalent and diverse in oral and gut, which are mainly related to the *TACK superphylum* and *Euryarchaeota*, especially methanogenic archaea 177. However, there are very few studies to elucidate how archaea regulate cancer treatment up to now.

## Future directions

The ecological imbalance within the individual appears to be a precursor to tumorigenesis, and maintaining an optimal composition of the microbial community is potentially the key to preventing tumorigenic events. Scientists are currently working to enhance treatment response and eliminate treatment-related toxicity by modulating the gut microbiota. Microbes can be modified through the management of FMT, probiotics, diet and lifestyle changes, and targeted regulation using customized antibiotic therapy or bacteriophages. In the future, it is likely that we will be able to combine pharmacogenomic analysis with customized microbiota or their specific metabolites to develop more precise and personalized cancer treatments. Moreover, we need to focus on the regulation of gut microbes, which requires a better understanding of the carcinogenic mechanisms. At present, the carcinogenic mechanisms of the microbiome have only been confirmed as causal in some GI cancers, and more plausible mechanisms need to be confirmed by more experimental and experiential evidence, including systematic reviews, high-quality randomized controlled trials and high-powered cohort studies. Future preclinical studies should use appropriate animal models to elucidate the role of microbes in the occurrence, progression and therapeutic response of GI cancer. These studies should be followed up with translational studies to meet the challenge of individualized treatment. With the increasing depth and breadth of research in this field, the microbiota will become an important part of cancer prevention and treatment. More large-scale and international cohort studies need to be carried out in the future, not only cross-sectional studies but also prospective longitudinal cohort studies. Currently, however, it is critical to carry out observational research and campaign for interventional research, combined with multi-omics approaches to carry out deep phenotyping of the microbiome, to make the transition from correlation to causal research.

## Conclusions

Numerous studies have provided evidence to support the concept that manipulation of the gut microbiome can be used as an adjunct strategy to improve the outcome of cancer treatment, although many of these studies are still in the early stages. The effect of anticancer drugs depends, to a large extent, on the gut microbiota, which can affect the response of intestinal and extraintestinal organs to anticancer chemotherapy. The composition of the gut microbiota can also be used as a potential biomarker for tumorigenesis and a target for cancer management. It can shed light on new directions for cancer prevention and the development of personalized treatment strategies. The interaction among host, drug and gut microbiota is complex; therefore, a personalized approach will be necessary for precision treatment. Additionally, the prevalence of excess body weight resulting in dysbiosis increase the risk of various cancers, thus, a balanced low-fat, low sugar, high fiber diet consisting of unprocessed foods and supplementation of safe probiotics and prebiotics have the potential for prevention of cancer, improving the efficacy of cancer treatment, and reducing adverse effects of anticancer drugs.

## Figures and Tables

**Table 1:** Gut microbiota involved in therapeutic responses and side-effects of GI cancer

Treatment	Gut Microbiota	Cancer type	Drug	Response/effect	Ref.
Response effect in chemotherapy	*F. nucleatum*	CRC	5-Fu Oxaliplatin	poor	109,110,111
		ESCC	CDDP Docetaxel		
	*Gammaprotect-obacteria*	CRC	Gemcitabine	poor	112
	*Clostridium*	LARC		poor	119
	*Enterococcus hirae*	CRC	CTX	favorable	115
	*Barnesiella intestinihominis*	CRC	CTX	favorable	115
Response effect In ICIs	*Prevotella*	CRC	anti-PD-1/PD-L1	favorable	143
	*Ruminococcaceae*	CRC	anti-PD-1/PD-L1	favorable	143
	* Lachnospiraceae*	CRC	anti-PD-1/PD-L1	favorable	143
	*Eubacterium*	CRC	anti-PD-1/PD-L1	favorable	143
	*Lactobacillus*	CRC	anti-PD-1/PD-L1	favorable	143
	*Streptococcus*	CRC	anti-PD-1/PD-L1	favorable	143
	*Bifidobacterium pseudolongum*	CRC	anti-CTLA-4/anti-PD-L1	favorable	149
Therapeutic side-effect	*Lactobacillus*		oxaliplatin	favorable	113
	*Roseburia*		radiotherapy	poor	123
	*Clostridium IV*		radiotherapy	poor	123
	*Faecalibacterium*		radiotherapy	poor	123
	*Lactobacillus rhamnosus*		radiotherapy	favorable	125
	*Lachnospiraceae*		radiotherapy	favorable	126
	*Enterococcaceae*		radiotherapy	favorable	126

## Abbreviations

B-HCC: hepatitis B virus-related HCC
BIRC3: baculoviral IAP repeat containing 3
*B.fragilis*: *Bacteroides fragilis*
*B. Clausii*: *Bacillus Clausii*
COX-2: cyclo-oxygenase 2
CTX: Cyclophosphamide
CTLA: cytotoxic T lymphocyte-associated antigen
*C. butyricum*: *Clostridium butyricum*
DCs: dendritic cells
DCA: deoxycholic acid
EAC: esophageal adenocarcinoma
*E.coli*: *Escherichia coli*
*E.hirae*: *Enterococcus hirae*
ESCC: esophageal squamous cell carcinoma
ETBF: Enterotoxigenic *B. fragilis*
EMT: epithelial-mesenchymal transformation
E-cadherin: epithelial-cadherin
FMT: fecal microbiota transplant
*F.nucleatum*: *Fusobacterium nucleatum*
FXR: farnesoid X receptor
*H. pylori*: *Helicobacter pylori*
GERD: gastroesophageal reflux disease
GI: Gastrointestinal
ICIs: immune checkpoint inhibitors
LPS: lipopolysaccharide
LARC: locally advanced rectal cancer
LTA: lipoteichoic acid
MDSCs: myeloid-derived suppressor cells
NGS: next-generation sequencing
NBNC-HCC: non-HBV non-hepatitis-C-virus- associated HCC
ROS: reactive oxygen species
SCFAs: short-chain fatty acids
SASP: senescence-associated secretory phenotype
*S. cerevisiae*: *Saccharomyces cerevisiae*
TLR: toll-like receptors
TGF: transforming-growth-factor
Tregs: regulatory cells
TNF-α: tumor necrosis factor-α
TAMs: tumor-associated macrophages
*P. gingivalis*: *Porphyromonas gingivalis*
PDAC: pancreatic ductal adenocarcinoma
PD-L1: programmed death ligand 1
UGT: UDP-glucuronosyltransferase
PAR1b: partitioning-defective 1b
*P. micra*: *Parvimonas micra*
NOX1: nicotinamide adenine dinucleotide phosphate oxidase 1
RASAL2: ras protein activator like 2
TUDCA: tauroursodeoxycholic acid
PSRs: plasma-stool ratios
CDCA: chenodeoxycholic acid
ILC2: type 2 innate lymphocytes
MMP9: matrix metalloproteinase 9
VEGFA: vascular endothelial growth factor A
CRC: colorectal cancer
HCC: hepatocellular carcinoma
NASH: non-alcoholic steatohepatitis
NAFLD: non-alcoholic fatty liver disease
NF: nuclear factor
O. viverrini: *Opisthorchis viverrini*
*K. pneumonia*: *Klebsiella pneumonia*
HDAC: histone deacetylase
PMT: photo-controlled bacterial metabolite therapy
Nb2C: niobium carbide
TILs: tumor infiltrating lymphocytes
